# Crucial Mutations of Spike Protein on SARS-CoV-2 Evolved to Variant Strains Escaping Neutralization of Convalescent Plasmas and RBD-Specific Monoclonal Antibodies

**DOI:** 10.3389/fimmu.2021.693775

**Published:** 2021-08-17

**Authors:** Chengchao Ding, Jun He, Xiangyu Zhang, Chengcheng Jiang, Yong Sun, Yuqing Zhang, Qingqing Chen, Hongliang He, Wenting Li, Jiajia Xie, Zhirong Liu, Yong Gao

**Affiliations:** ^1^The First Affiliated Hospital of USTC, Division of Life Sciences and Medicine, University of Science and Technology of China (USTC), Hefei, China; ^2^Department of Microbiology, Anhui Provincial Center for Disease Control and Prevention, Hefei, China

**Keywords:** SARS-CoV-2, crucial mutation, cross-neutralization, monoclonal antibody, spike protein

## Abstract

Small number of SARS-CoV-2 epidemic lineages did not efficiently exhibit a neutralization profile, while single amino acid mutation in the spike protein has not been confirmed in altering viral antigenicity resulting in immune escape. To identify crucial mutations in spike protein that escape humoral immune response, we evaluated the cross-neutralization of convalescent plasmas and RBD-specific monoclonal antibodies (mAbs) against various spike protein-based pseudoviruses. Three of 24 SARS-CoV-2 pseudoviruses containing different mutations in spike protein, including D614G, A475V, and E484Q, consistently showed an altered sensitivity to neutralization by convalescent plasmas. A475V and E484Q mutants are highly resistant to neutralization by mAb B38 and 2-4, suggesting that some crucial mutations in spike protein might evolve SARS-CoV-2 variants capable of escaping humoral immune response.

## Introduction

Since the coronavirus disease 2019 (COVID-19) pandemic is still spreading globally, specific antibody therapy and vaccine have now been approved to combat the causative agent SARS-CoV-2 (1, 2). Direct transfusion of specific SARS-CoV-2 antibodies, i.e., passive immunization, allows the individual to acquire immediate but short immunity, while the COVID-19 vaccine is able to elicit long-lasting immunity in the vaccinees. Both of the two strategies have showed a remarkable effect on the control of the epidemic and/or the recovery of the patients (3, 4).

Different from other RNA viruses, the non-structural protein nsp14 of SARS-CoV-2 has 3’-5’ exonuclease activity correcting the nucleotide mismatches occurred in the process of viral genome transcription (5, 6). Therefore, the overall mutation frequency of SARS-CoV-2 genome is lower than that of HIV-1 and influenza viruses. However, the recent emergence of a new circulating mutant strain has raised public concern about the protection efficiency of the COVID-19 vaccine and antibody therapy. Single amino acid mutation in the spike protein could affect the physicochemical property and structure of the protein, which subsequently alters the affinity of the receptor binding domain (RBD) with the viral receptor, human angiotensin-converting enzyme 2 (hACE2) (7, 8). Importantly, the emergence of these amino acid mutations might result in a reduced neutralization ability of the immune serum from vaccinees or the existing antibodies in recovered COVID-19 patients, causing a new wave of the epidemic.

The mutation in the spike protein not only affects the replication capacity and infectivity of the virus, but also impacts the host immune response. During the SARS epidemic in 2003, the single amino acid mutation D480A/G in the RBD domain of SARS-CoV spike protein gradually became dominant, and subsequent study confirmed that this mutation occurred in a critical site and enabled the mutant escaping neutralizing antibodies (9). In contrast, early in the COVID-19 pandemic, SARS-CoV-2 spike mutation D614G rapidly became globally dominant. Several studies demonstrated that the D614G mutation enhanced the replication of the mutated virus in the lung epithelial cells (10). This variant has a higher infectiousness due to its higher viral load in the respiratory secretions. However, a recent study indicated that the neutralizing activity of immune sera from vaccinees against G614 variant was better than the original one, and revealed that the structure of the G614 spike had a more open ACE2 binding site in the RBD region (11). In addition, several recent studies have focused on the neutralizing activity of vaccine-elicited humoral immunity against new circulating mutant lineages, including B.1.1.7 (United Kingdom, bearing mutations 69–70 del, 144 del, N501Y, A570D, D614G, P681H, T716I, S982A, and D1118H in the spike protein), B.1.429 (United States, bearing mutations S13I, W152C, L452R, and D614G in the spike protein), B.1.351(South Africa, bearing mutations D80A, D215G, K417N, E484K, N501Y D614G, and A701V in the spike protein), and P.1 and P.2 (Brazil, bearing certain mutations E484K, D614G, and V1176F, etc. in the spike protein) (12, 13). Amongst these mutants, the neutralization susceptibility of B.1.351 was significantly decreased to the immune sera from vaccinees (14–16). However, crucial single amino acid mutations that altered the antigenicity of the spike protein have not been fully explored, since natural variants generally containing multiple mutations at different sites.

Although the mutation frequency of SARS-CoV-2 is lower than that of other RNA viruses without a correction mechanism, the rapid global spread of SARS-CoV-2 and large amounts of infections still produce sufficient natural mutations for the virus. Spike protein is the main antigen that induces protective immune responses, thus, become the main target of neutralizing antibodies and currently used vaccines (17, 18). Antigenic drift may occur in the glycoprotein of the mutant virus, thus, antibodies derived from the original strain might only afford a partial cross-neutralizing effect against different variants. Obviously, it is important to keep a close eye on the evolution and mutation of the spike protein during transmission of the virus. To identify the crucial amino acid mutations that alter the antigenicity of the virus, we generated 24 pseudoviruses with different individual spike mutations that occurred naturally and frequently in the epidemic to determine their possible resistance to specific immune responses, and found that two variants with a crucial single amino acid mutation on the spike protein were capable of escaping the neutralization by convalescent plasma and RBD-specific monoclonal antibodies.

## Methods

### Human Samples

A total of 20 recovered COVID-19 patients from the First Affiliated Hospital of USTC were enrolled in this study. All participants provided written informed consent. The convalescent plasma samples were obtained at 3–5 months after the initiation of the disease.

### Construction of Plasmids and Cell Lines

SARS-CoV-2 surface glycoprotein gene (GenBank: MN_908947) with C-terminal 19 amino acids deletion was codon-optimized for *Homo sapiens* and cloned into the eukaryotic expression plasmid pcDNA3.1(+) between the *Hind* III and *BamH* I sites to generate the envelope expression plasmid pcDNA3.1(+)-Opt-S. The lentiviral packaging plasmid pNL4-3 Luc+R-E- carrying an Env-defective and luciferase-expressed HIV-1 genome was generously gifted by Binlian Sun, Jianghan University. To simulate the virus entry assay *in vitro*, HEK293T cell line exogenously expressing hACE2 (HEK293T/hACE2) was constructed for pseudovirus neutralization assay in this study. HEK 293F cell line was generously gifted by Tengchuan Jin, University of Science and Technology of China. HEK293T and HEK293T/hACE2 cells were maintained in high glucose Dulbecco’s Modified Eagle Medium (Gibco) supplemented with 10% FBS (ExCell), penicillin (100 IU/ml), and streptomycin (100 μg/ml) in a 5% CO_2_ environment at 37°C and passaged every 3 days. HEK293F cells were maintained in Chemically Defined Medium (Union-Biotech Co. Ltd, Shanghai) supplemented with penicillin (100 IU/ml) and streptomycin (100 μg/ml) in 37°C shaker at 120 rpm with 5% CO_2_.

### Selection of Spike Mutation and Production of Corresponding Pseudoviruses

**Figure 1:**
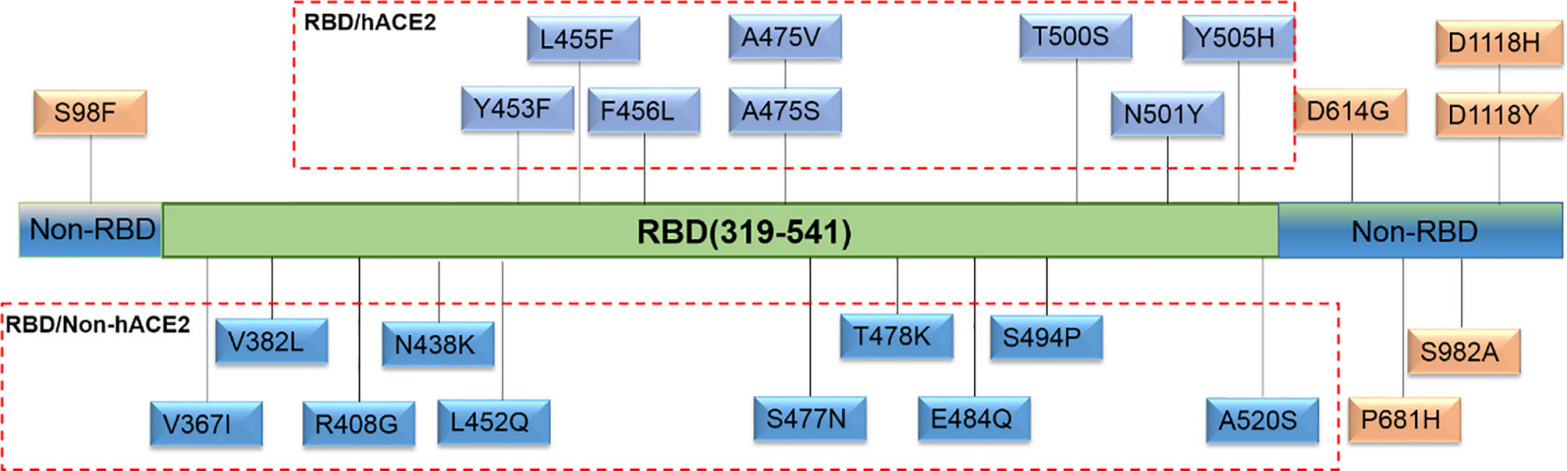
Natural and high-frequency amino acid mutations of the spike protein selected in this study. Twenty-four single amino acid mutations were located in three regions. Eight mutations (Y453F, L455F, F456L, A475V, A475S, T500S, N501Y, and Y505H) were in the RBD and hACE2 interaction region (RBD/hACE2); 10 mutations (V367I, V382L, R408G, N438K, L452Q, S477N, T478K, E484Q, S494P, and A520S) were in the RBD region but no interaction with hACE2 (RBD/Non-hACE2); 6 mutations (S98F, D614G, P681H, S982A, D1118H, and D1118Y) were not in the RBD region (Non-RBD).

For the selection of spike mutations that occurred naturally and frequently, we retrieved a total of 24 sequences of S gene in the RBD and hACE2 interaction region (RBD/hACE2, n = 8), RBD region but no interaction with hACE2 (RBD/Non-hACE2, n = 10), and Non-RBD region (Non-RBD, n = 6) in the GISAID database up to March 1, 2021. All selected single amino acid mutations in the above-mentioned regions are shown in **Figure 1**. To generate the plasmids encoding various spike mutants, pcDNA3.1(+)-Opt-S plasmid was used as a template to perform site-directed mutagenesis through recombinational PCR amplification. The resultant PCR product was then digested using *DpnI* restriction endonuclease to exclude the template plasmid. The digested product was transformed into Stbl3 competent cells, and single clones were selected and sequenced.

Pseudoviruses with spike mutations were produced by the co-transfection of HEK293T cells with pNL4-3 Luc+R-E- and plasmids encoding various spike mutations, while Wuhan reference pseudovirus was produced with pNL4-3 Luc+R-E- and pcDNA3.1(+)-Opt-S. Briefly, 24 hours prior to transfection, HEK293T cells were prepared at 7 × 10^6^ cells/T_75_ and cultured at 37°C with 5% CO_2_. The two plasmids (50 mg, 1:1 copy numbers) were then transfected into the HEK293T cells using polyetherimide (PEI). The supernatants were harvested at 48 h post transfection, centrifuged at 300 × g for 10 min, passed through 0.45 μm filter, and stored at -80°C in 1 mL aliquots until use.

### Expression and Purification of RBD-Specific mAbs

The amino acid sequences of three published SARS-CoV-2 mAbs (mAb CC12.1, mAb 2-4, and mAb B38) were downloaded from the PDB (protein data bank) database (http://www.rcsb.org/). Each antibody DNA sequence transformed from the amino acid sequence was codon-optimized for human cells. The resultant sequences of paired heavy and light chain variable regions were then synthesized (General Biosystems Co. Ltd, Anhui, China) and separately cloned into expression vectors containing human IgG1 constant regions which were co-transfected into HEK293F cells using PEI and cultured in a 37°C shaker at 120 rpm with 5% CO_2_. The culture supernatant was collected 5 days post-transfection and the produced mAbs were purified using the Protein A sepharose column (GE Healthcare) and eluted with buffer containing 20 mM Na_2_HPO_4_, 150 mM NaCl, and substitution with PBS by centrifugation with 10 kDa molecular weight cut-off membrane centrifugal filter (Millipore).

### Pseudovirus Neutralization Assay

**Figure 2:**
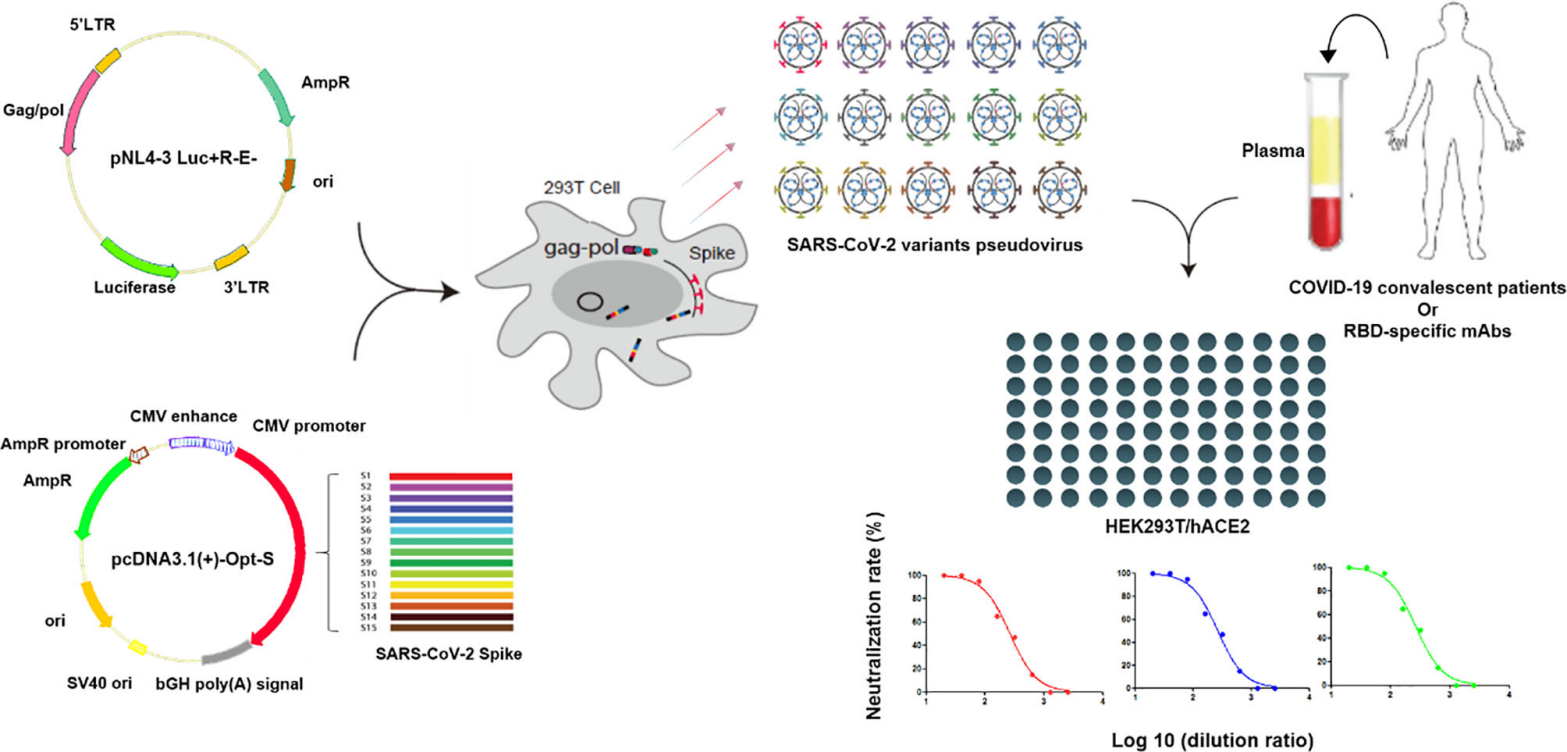
Illustration of pseudovirus production and neutralization assay. Pseudoviruses were produced by the co-transfection of HEK293T cells with pNL4-3 Luc+R-E- and pcDNA3.1(+)-Opt-S plasmids expressing spike proteins. Neutralization assay was performed by incubating the pseudovirus with diluted convalescent plasmas or mAbs, and the neutralization rates were detected in the HEK293T/hACE2 cells.

Pseudovirus neutralization assay was performed by incubating the pseudovirus with serial dilutions of convalescent plasma or purified mAbs and detecting the luciferase expression level in target cells. Briefly, HEK293T/hACE2 cells were seeded in 96-well cell plates at 10,000 cells/well in the culture medium 24 h prior to detection. The convalescent plasmas were 2-fold serially diluted in the culture medium with the initial dilution of 1:20 (dilution range of 1:20–1:2,560). Purified mAbs were 5-fold serially diluted in the culture medium with the initial dilution of 10 μg/mL (concentration range of 10 μg/mL–1.28×10^-4^ μg/mL). A total of 50 µL of pseudovirus with the values of relative luminescence unit (RLU) at approximately 1.0×10^5^ was incubated with diluted plasma or mAb at 37°C for 1 h, which were then added to HEK293T/hACE2 cells. After 48 h of incubation at 37°C with 5% CO_2_, culture supernatants were removed and the values of RLU were measured by the Britelite plus Reporter Gene Assay System (PerkinElmer). The illustration of pseudovirus production and neutralization assay is shown in **Figure 2**.

### Statistical Analysis

The NTIC50 or IC50 was defined as the dilution (plasma dilution to NTIC50, mAb concentration to IC50) at which the RLU values were reduced by 50% compared with the pseudovirus control wells calculated by the GraphPad Prism 8 (GraphPad Software). The difference of geometric mean titer (GMT) in two independent groups was tested for statistical significance with a Mann-Whitney U test in the GraphPad Prism 8. The difference of GMT in two paired groups was tested for statistical significance with a Wilcoxon matched-pairs signed rank test in the GraphPad Prism 8. Differences were considered statistically significant at *p < 0.05, **p < 0.01, or ***p < 0.001.

## Results

### Neutralizing Antibody Responses Against the Wuhan Reference Pseudovirus in Convalescent Plasma

**Figure 3:**
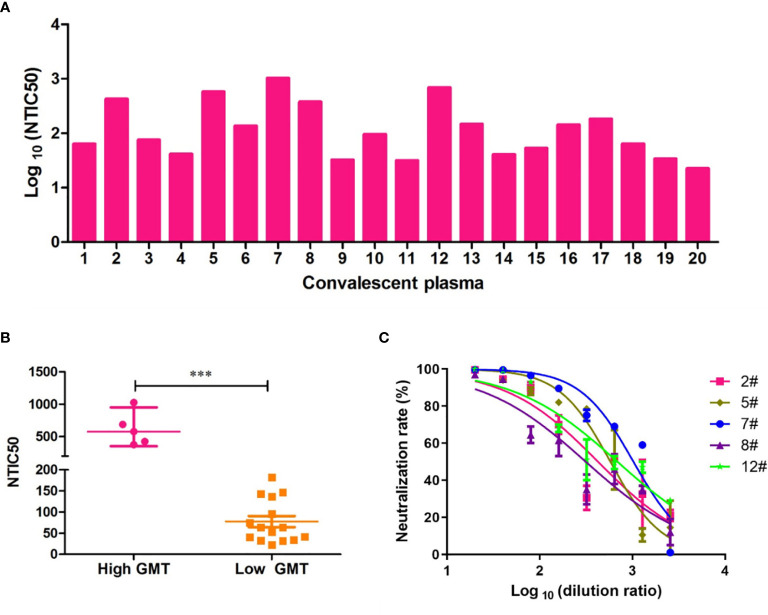
Neutralization titers of convalescent plasmas against pseudovirus bearing the Wuhan reference spike protein. **(A)** Neutralization titer of 50% Wuhan pseudovirus inhibition (NTIC50) of 20 plasma samples from convalescent COVID-19 patients. **(B)** Clustering analysis in higher and lower GMT convalescent plasma samples. The difference of GMT in two groups was tested for statistical significance with a Mann-Whitney U test in the GraphPad Prism 8. Differences were considered statistically significant at ***p < 0.001. **(C)** Neutralization curves of the five convalescent plasma samples (2#, 5#, 7#, 8#, and 12#) that show a higher neutralization titer than others against the Wuhan pseudovirus.

To assess the neutralizing antibody responses against the Wuhan reference pseudovirus in the plasmas from COVID-19 recovered patients, we included 20 patients from the First Affiliated Hospital of USTC. The time between the recovery to sample collection was 3–5 months. As shown in **Figure 3A**, NTIC50 of 20 convalescent plasma samples ranged from 1.5 to 3.0 of Log 10, with a GMT of neutralizing antibodies 110.2 (95% CI 64.4–188.7). As shown in **Figure 3C**, the neutralization curves of five samples (2#, 5#, 7#, 8#, and 12#) show a significantly higher GMT (579.4, 95% CI 353.0–950.9, **Figure 3B** left) than others (63.4, 95% CI 44.0–91.3, **Figure 3B** right) against the Wuhan reference pseudovirus. We deduced that convalescent plasma with a higher GMT might bear more abundant neutralizing antibodies against SARS-CoV-2. Thus, five plasma samples with a higher GMT were then used to explore the impact on humoral immune response against pseudovirus bearing the various mutated spike proteins.

### Neutralizing Antibody Responses Against Variant Pseudoviruses in Five High GMT Convalescent Plasma Samples

**Figure 4:**
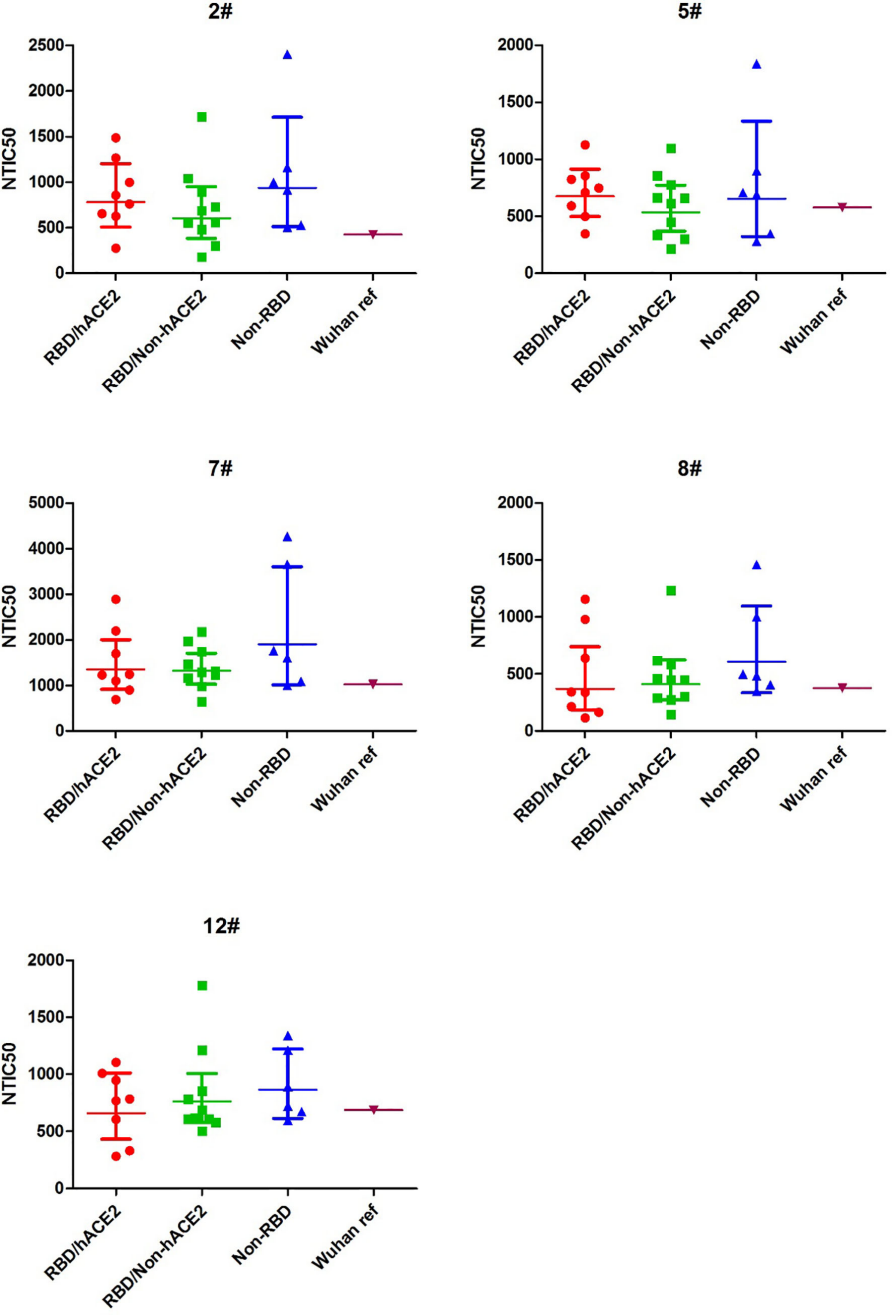
NTIC50 against pseudovirus bearing the various spike mutants for the five convalescent plasma samples (2#, 5#, 7#, 8#, and 12#) that show a higher neutralization titer against the Wuhan reference pseudovirus. The difference of GMT in different regions was tested for statistical significance with a Mann-Whitney U test in the GraphPad Prism 8.

To explore the difference of the neutralizing antibody responses against variant pseudoviruses, we constructed a total 24 SARS-CoV-2 spike protein pseudoviruses with a single amino acid mutation in RBD/hACE2 (n = 8), RBD/Non-hACE2 (n = 10), or Non-RBD (n = 6) regions. All selected single amino acid mutations in the above-mentioned regions are shown in **Figure 1**. Since five plasma samples showed a higher GMT in the convalescent plasma neutralization assay, we next determined how these plasma samples reacted to the variant pseudoviruses. The illustration of pseudovirus production and neutralization assay is shown in **Figure 2**. The neutralization curves of the five samples (2#, 5#, 7#, 8#, and 12#) against variant pseudoviruses are shown in **Supplementary Figure 1**. When the GMT values of variants with mutations in each region were pooled together to separately analyze the neutralizing activity in the five samples, no significant differences were observed (shown in **Figure 4**). However, the GMT of the Non-RBD group was slightly higher than that in other two groups (i.e., RBD/Non-hACE2 and Non-RBD).

### Relative Neutralization Activity of Variant Pseudoviruses

**Figure 5:**
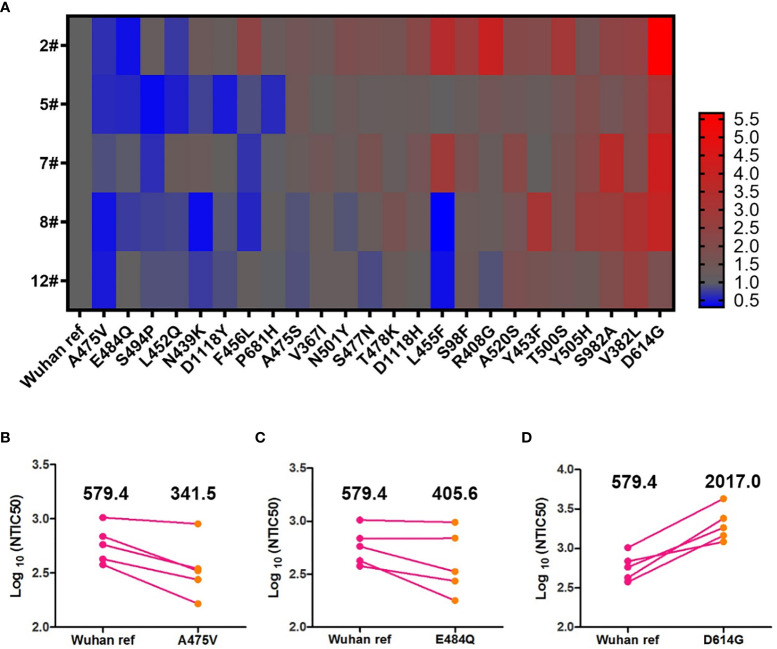
Neutralization analysis of convalescent plasmas (n = 5) against the Wuhan reference and variant pseudoviruses. **(A)** Heat map of the relative neutralizing activities of the five convalescent plasma samples against pseudoviruses bearing the variant spike proteins compared to the Wuhan reference pseudovirus. (Blue denotes reduction; red denotes increase). Paired neutralization analyses of five convalescent plasma samples against the Wuhan reference and variant pseudoviruses of A475V, E484Q, and D614G are shown in **(B–D)**, respectively. GMT is shown above each graph. The difference of GMT was tested for statistical significance with a Wilcoxon matched-pairs signed rank test in the GraphPad Prism 8.

Antigenic drift may occur in the glycoprotein of mutant viruses in the global pandemic in a short time. To investigate the immune escape of variants that occurred naturally, we analyzed the NTIC50 of five high GMT convalescent plasma samples against 24 variant pseudoviruses. NTIC50 of variant pseudoviruses relative to the Wuhan reference pseudovirus are shown in **Figure 5A**. Three variant pseudoviruses with a single amino acid mutation including A475V, E484Q, and D614G consistently altered the neutralization sensitivity to the five convalescent plasma samples. When compared with the Wuhan reference pseudovirus (GMT = 579.4), paired neutralization analysis showed that the convalescent plasma reduced the neutralizing activity against A475V (GMT = 341.5, **Figure 5B**) and E484Q (GMT = 405.6, **Figure 5C**) variants but increased the neutralizing activity against the D614G variant (GMT = 2,017.0, **Figure 5D**). However, when the GMT values of these three variant pseudoviruses were analyzed for the relative neutralizing activity to the Wuhan reference pseudovirus, no significant differences were observed (Wuhan reference *vs.* A475V, *p* = 0.0625; Wuhan reference *vs.* E484Q, *p* = 0.1250; and Wuhan reference *vs.* D614G, *p* = 0.0625).

### Neutralization Analysis of RBD-Specific mAbs Against A475V and E484Q Variant Pseudoviruses

**Figure 6:**
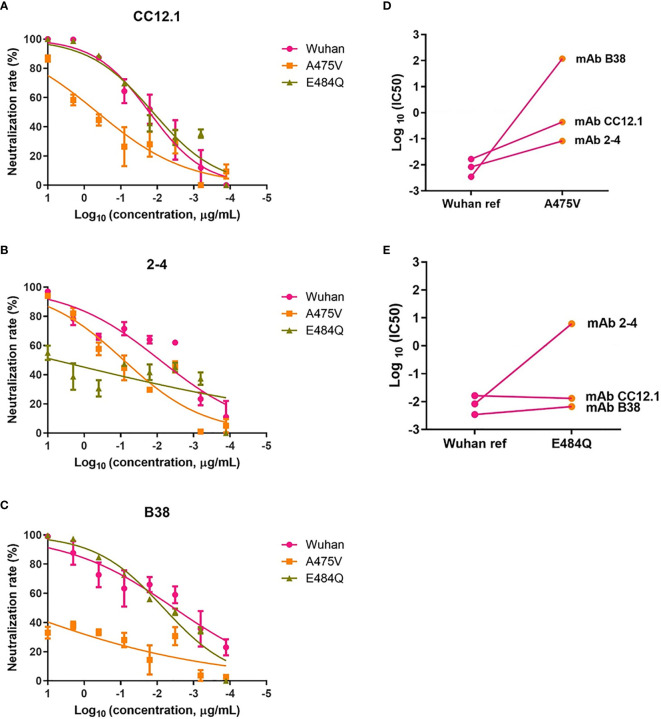
Neutralization analysis of RBD-specific mAbs (n = 3) against pseudoviruses with the Wuhan reference spike protein and variant spike protein A475V or E484Q. Neutralization curves of three mAbs (CC12.1, **(A)**; 2-4, **(B)**; B38, **(C)** against the Wuhan reference pseudovirus, A475V variant pseudovirus, and E484Q variant pseudovirus in the HEK293T/hACE2 cells. Paired neutralization analyses of three mAbs against the Wuhan reference and variant pseudoviruses of A475V and E484Q are shown in **(D, E)**. The 50% pseudovirus inhibition concentration (IC50) of mAb was defined as the concentration at which the RLU values were reduced by 50% compared with the pseudovirus control wells, which was calculated in the GraphPad Prism 8 (GraphPad Software).

Previous study indicated that the plasma from convalescent COVID-19 patients was rich in SARS-CoV-2 specific polyclonal antibodies (19). The results of the relative neutralizing activity analysis suggested that A475V and E484Q might decrease the sensitivity of the variant to the polyclonal antibodies from the convalescent plasma. Therefore, we finally explored whether these mutations impact the neutralizing activity of RBD-specific mAbs. The neutralization curves of three RBD-specific mAbs (CC12.1, 2-4, and B38) against the Wuhan reference pseudovirus, A475V and E484Q variant pseudoviruses are respectively shown in **Figures 6A–C**, respectively. The neutralizing activity against two variant pseudoviruses relative to the Wuhan reference pseudovirus is shown in **Figures 6D, E**. We found that CC12.1 was effective in neutralizing both A475V and E484Q variant pseudoviruses. It is notable that antibody B38 was unable to effectively neutralize A475V variant pseudovirus (**Figure 6D**) while the neutralizing activity of antibody 2-4 against E484Q variant pseudovirus was also decreased nearly 1,000-fold (**Figure 6E**).

## Discussion

In the convalescent plasma, we speculated that samples with a high neutralization titer might contain more abundant specific neutralizing antibodies, and novel high-affinity neutralizing antibodies or broadly neutralizing antibodies against SARS-CoV-2 variants are more likely to emerge in these individuals. Therefore, in the present study, we first used the plasma samples with a high neutralization titer to explore the cross-neutralization against the variants with different mutations in the spike protein. We found that there were slight differences of the neutralizing activity among the three groups (RBD/hACE2, RBD/Non-hACE2, and Non-RBD) of spike mutants but were insignificant (**Figure 4**). It was accepted that the mutations located in the RBD/hACE2 region more likely alter the viral neutralization reactivity to antibodies (20). Our results of the neutralization of the convalescent plasma against different spike variants indicated that mutations in either RBD/hACE2 or RBD/Non-hACE2 interaction regions slightly decreased the neutralizing activity of the convalescent plasma against the corresponding pseudoviruses. The reason might lie in the fact that there are multiple neutralizing antibodies in the patients and a few mutations in the virus might not sufficiently enable the virus escape from immune attack. On the other hand, since the mutation in the non-RBD and ACE2 interaction region resulted in a decreased neutralization sensitivity, we should also pay attention to the mutations in the entire spike protein region in order to fully monitor the possible mutations causing immune escape.

In paired neutralization analysis of the Wuhan reference and D614G variant pseudoviruses, we found that the D614G variant pseudovirus was more sensitive to neutralization by the convalescent plasma (**Figure 5**). Previous study revealed that the percentage of RBD “up” conformation in the G614 spike was higher than that in the D614 spike (21), which might explain that the D614G variant is more infectious while more sensitive to neutralization. Therefore, we suspect that D614G and possibly other sensitive variants might not be challenges for current COVID-19 vaccines. However, we also found that certain variants with a single amino acid mutation (e.g., A475V and E484Q) might be able to escape neutralization by plasma from COVID-19 recovered patients. The decreased neutralization activities highlight that we should absolutely focus on the effect of vaccines against these variants that are resistant to humoral immunity, especially when these mutations occur simultaneously. Additionally, we speculate that the decreased neutralization activities of A475V and E484Q might be due to their resistance to some mAbs. The plasma neutralization assay indicated that epidemic variants including either of the mutant sites described above might indeed partially escape neutralization by humoral immunity.

Importantly, the aforementioned immune escape was further confirmed through the neutralization assay with RBD-specific mAbs. The neutralizing activity of mAb B38 against A475V mutant was reduced 10,000 folds when compared with the wild type virus (**Figure 6**), while the efficacy of mAb 2-4 in neutralizing the E484Q mutant also decreased up to 1,000 folds (**Figure 6**). We found that A475 is one of the direct interaction residues between antibody and RBD when reviewing the structural basis of mAb B38 in the original study (22). Similarly, a previous cryo-EM analysis of mAb 2-4 in complex with the spike trimer shows that E484 is also one of key sites binding to this specific antibody (23). The phenomenon of a decreased neutralization activity of some RBD-specific mAbs against RBD mutants suggests that cocktail therapy with multiple mAbs might be necessary in order to effectively suppress SARS-CoV-2 replication and to avoid immune escape. A potential limitation of this study lies in the lack of neutralization activity analysis with vaccine-induced plasma. However, a previous study indicated that neutralization activities consist between vaccination and convalescent sera (24). It should be noted that, compared with antibody therapy, it is much more comprehensive to develop a COVID-19 vaccine that can elicit broadly neutralizing antibodies against various variants other than specific ones against a few strains.

## Data Availability Statement

The raw data supporting the conclusions of this article will be made available by the authors, without undue reservation.

## Ethics Statement

The studies involving human participants were reviewed and approved by the ethics committee of the First Affiliated Hospital of USTC. The patients/participants provided their written informed consent to participate in this study.

## Author Contributions

JX, ZL, and YG designed the study. CD, JH, XZ, CJ, YS, YZ, QC, WL, and HH performed the experiments. CD analyzed the data. CD and YG wrote the manuscript. All authors contributed to the article and approved the submitted version.

## Funding

This work is supported by the Key Research and Development Project of Anhui Province (202104j07020042; 202104a07020032), Special Project for Emergency Scientific and Technological Research on New Coronavirus Infection (YD9110002001), Emergency Research Project of Novel Coronavirus Infection of Anhui Province (202004a07020002; 202004a07020004), Postdoctoral Research Foundation of China (2021M693076; 2020M670084ZX), the Fundamental Research Funds for the Central Universities (WK9110000166; WK9110000167), and the Hefei Comprehensive National Science Center.

## Conflict of Interest

The authors declare that the research was conducted in the absence of any commercial or financial relationships that could be construed as a potential conflict of interest.

## Publisher’s Note

All claims expressed in this article are solely those of the authors and do not necessarily represent those of their affiliated organizations, or those of the publisher, the editors and the reviewers. Any product that may be evaluated in this article, or claim that may be made by its manufacturer, is not guaranteed or endorsed by the publisher.
