# Supplementary material for: Crucial Mutations of Spike Protein on SARS-CoV-2 Evolved to Variant Strains Escaping Neutralization of Convalescent Plasmas and RBD-Specific Monoclonal Antibodies
Source: Front Immunol. 2021 Aug 17;12:693775. doi: 10.3389/fimmu.2021.693775 (PMC8416052; doi:10.3389/fimmu.2021.693775)
Supplement: Supplementary file 1 [file DataSheet_1.docx]

**Fig 1S. Neutralization curves of 5 samples (2#, 5#, 7#, 8# and 12#) against variant pseudoviruses.** Variant pseudoviruses with spike mutations located in RBD/hACE2 region (A), RBD/Non-hACE2 region (B) and Non-RBD region (C).

**
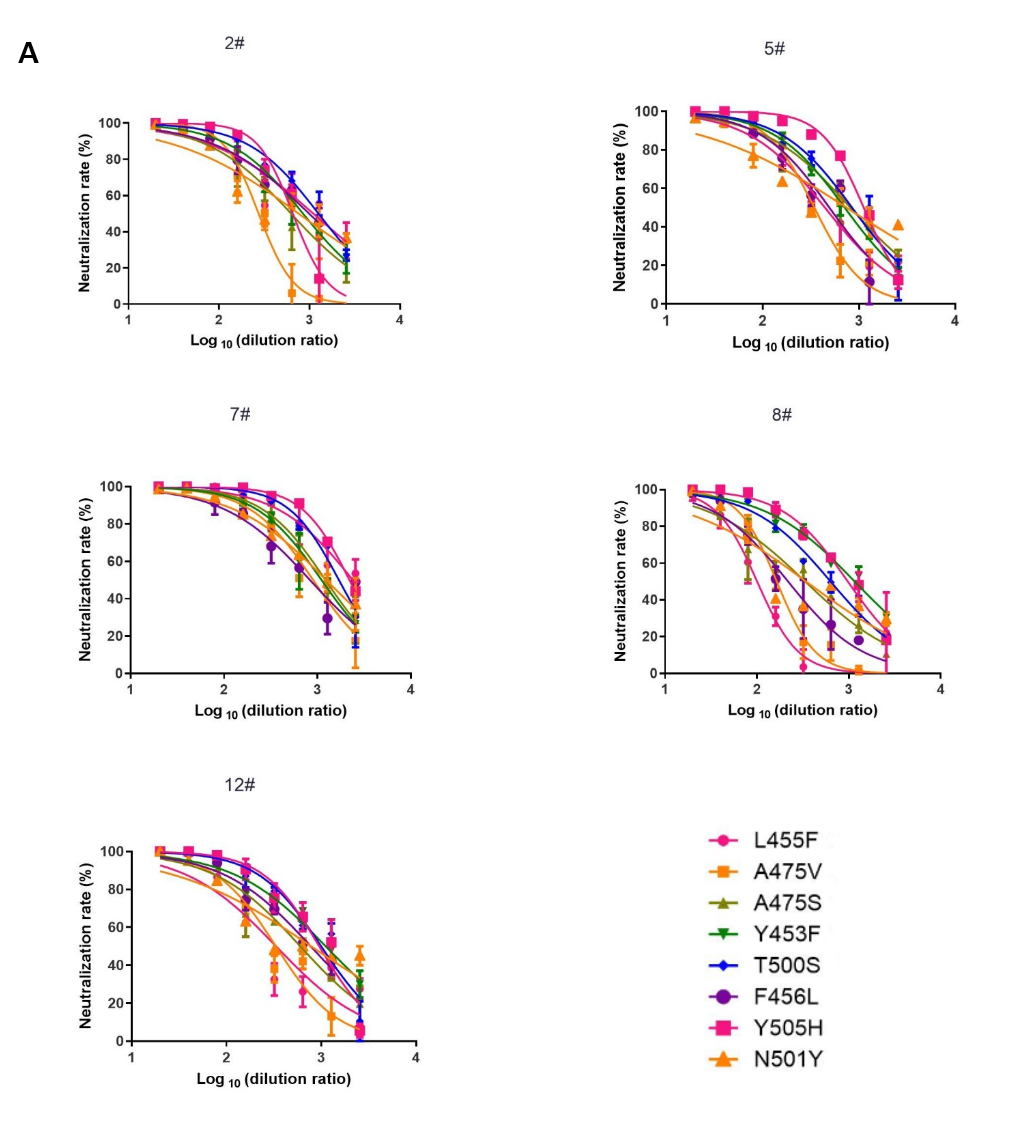

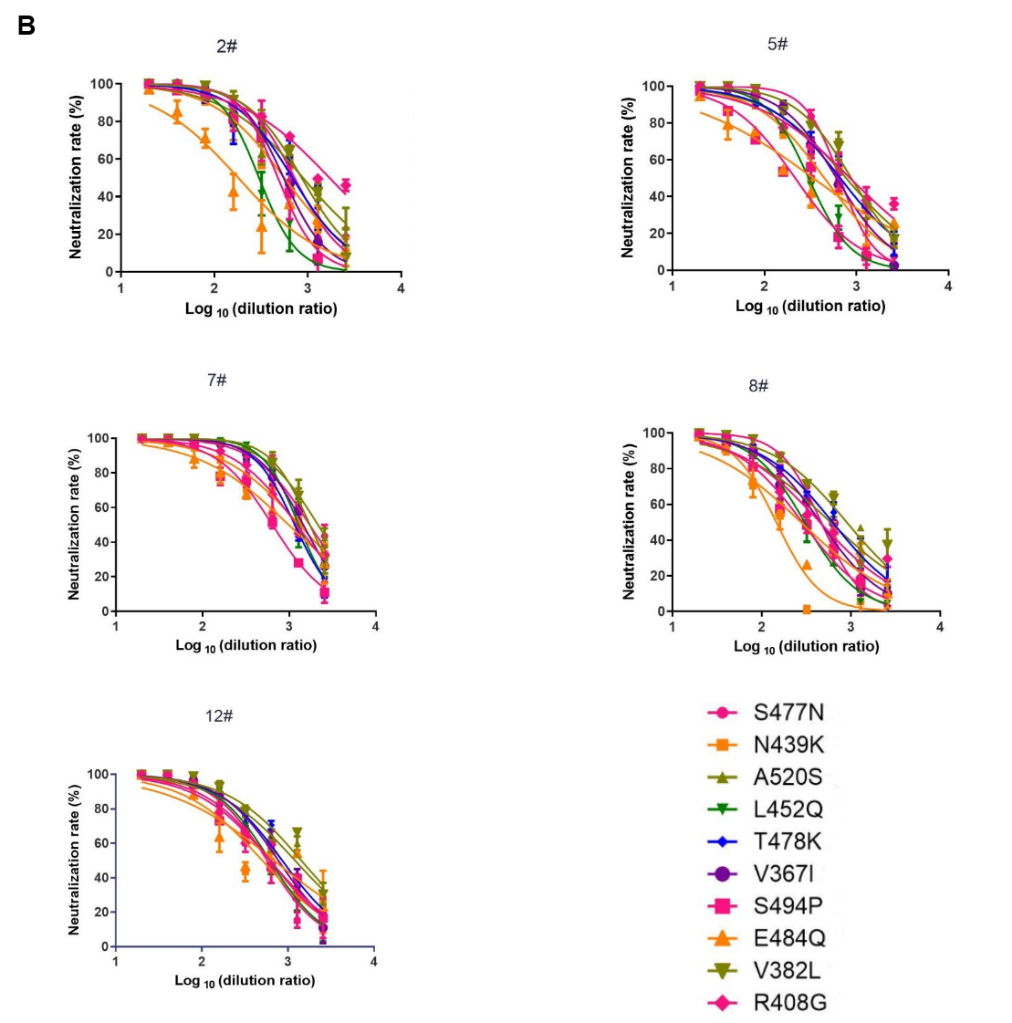

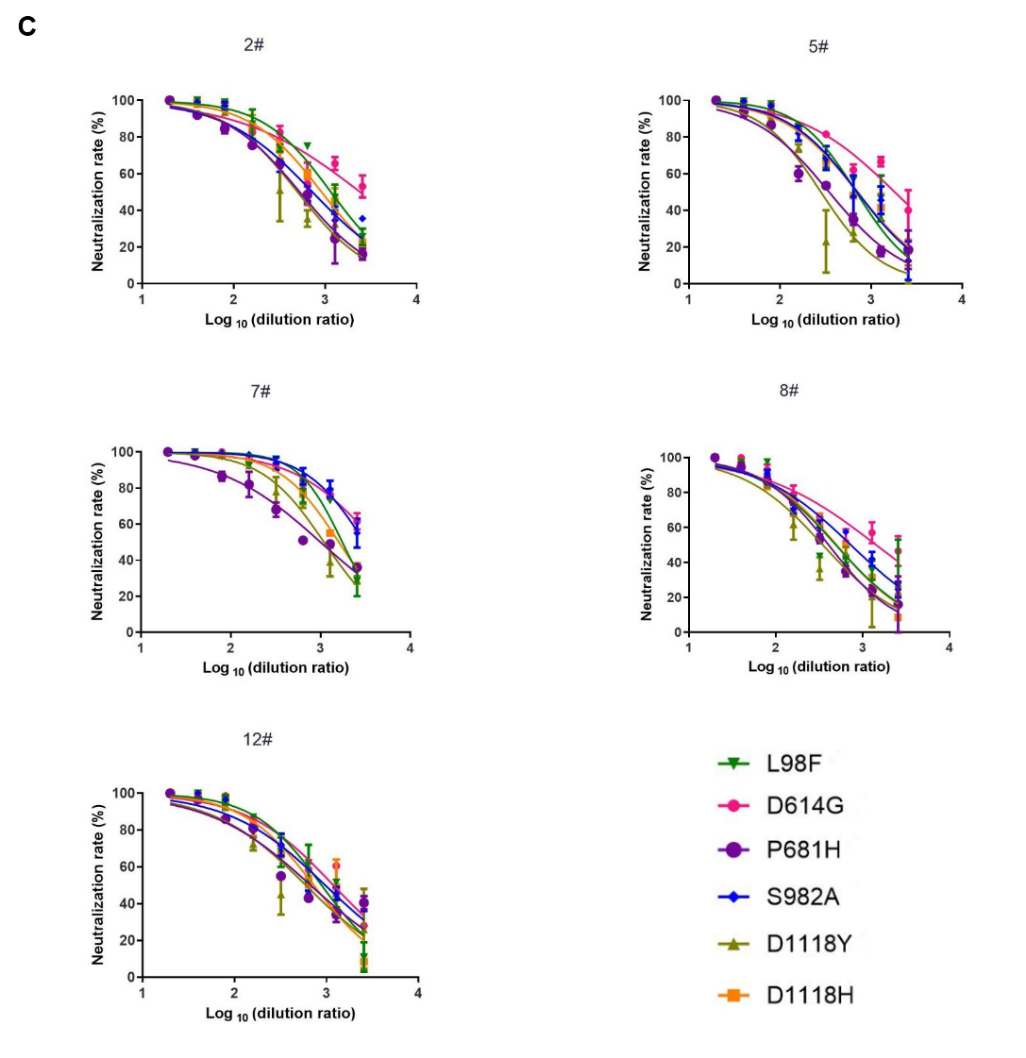
**
